# Assessing researchers’ capabilities, opportunities, and motivation to conduct equity-oriented dissemination and implementation research, an exploratory cross-sectional study

**DOI:** 10.1186/s12913-022-07882-x

**Published:** 2022-06-01

**Authors:** Ana A. Baumann, Eva N. Woodward, Rajinder Sonia Singh, Prajakta Adsul, Rachel C. Shelton

**Affiliations:** 1 Division of Public Health Sciences, Department of Surgery, Washington University School of Medicine, St. Louis, MO USA; 2 Center for Mental Healthcare and Outcomes Research, U.S. Department of Veterans Affairs, North Little Rock, AR USA; 3 Department of Psychiatry, University of Arkansas for Medical Sciences, Little Rock, AR USA; 4 South Central Mental Illness Research, Education and Clinical Center, U.S. Department of Veterans Affairs, North Little Rock, North Little Rock, AR USA; 5 Department of Psychiatry, University of Arkansas for Medical Sciences, Little Rock, AR USA; 6 Department of Internal Medicine, School of Medicine, University of New Mexico, Albuquerque, NM USA; 7 Department of Sociomedical Sciences, Columbia University, Mailman School of Public Health, New York, NY USA

**Keywords:** Equity, Dissemination and implementation research, Training, Capability, Motivation, Opportunity

## Abstract

**Background:**

A recent paradigm shift has led to an explicit focus on enhancing health equity through equity-oriented dissemination and implementation (D&I) research. However, the integration and bidirectional learning across these two fields is still in its infancy and siloed. This exploratory study aimed to examine participants’ perceived capabilities, opportunities, and motivations to conduct equity-oriented D&I research.

**Methods:**

We conducted an exploratory cross-sectional survey distributed online from December 2020 to April 2021. Participants were recruited at either D&I or health disparities-oriented conferences, meetings, through social media, or personal outreach via emails. Informed by the Capability, Opportunity, and Motivation Model (COM-B), the survey queried respondents about different aspects of engaging in and conducting equity-oriented D&I research. All analyses were conducted in SPSS Version 27.0.

**Results:**

A total of 180 participants responded to the survey. Most participants were women (81.7%), white (66.1%), academics (78.9%), and faculty members (53.9%). Many reported they were advanced (36.7%) or advanced beginners (27.8%) in the D&I field, and a substantial proportion (37.8%) reported being novice in D&I research that focused on health equity. Participants reported high motivation (e.g., 62.8% were motivated to apply theories, models, frameworks for promoting health equity in D&I research), but low capability to conduct equity-oriented D&I research (e.g., 5% had the information needed for promoting health equity in D&I research). Most participants (62.2%) reported not having used measures to examine equity in their D&I projects, and for those who did use measures, they mainly used individual-level measures (vs. organizational- or structural-level measures). When asked about factors that could influence their ability to conduct equity-oriented D&I research, 44.4% reported not having the skills necessary, and 32.2% stated difficulties in receiving funding for equity-oriented D&I research.

**Conclusions:**

Study findings provide empirical insight into the perspectives of researchers from different backgrounds on what is needed to conduct equity-oriented D&I research. These data suggest the need for a multi-pronged approach to enhance the capability and opportunities for conducting equity-oriented D&I work, such as: training specifically in equity-oriented D&I, collaboration between D&I researchers with individuals with expertise and lived experience with health equity research, funding for equity-oriented D&I research, and recognition of the value of community engaged research in promotion packages.

**Supplementary Information:**

The online version contains supplementary material available at 10.1186/s12913-022-07882-x.

## Contributions to the literature

This paper reports findings from an online survey with 180 participants that examined respondents’ perceived capabilities, opportunities and motivation to conduct equity-oriented D&I research.

While respondents reported high levels of motivation to engage in equity-oriented D&I research, many identified the need for specific trainings in how to conduct this work, including increasing knowledge of equity-focused frameworks, measures and skills in conducting equity-oriented D&I research, as well as greater opportunities for funding and collaborations in this area.

We highlight data-informed leverage points for promoting equity-oriented D&I research, including information for developing future training programs and funding opportunities.

## Background

The recent paradigm shift in the field of dissemination and implementation (D&I) science has been an explicit focus on enhancing health equity and reducing health disparities through equity-oriented D&I research [1, 2]. Equity-oriented D&I research occurs “when strong equity components—including explicit attention to the culture, history, values, assets, and needs of the community—are integrated into the principles, strategies, frameworks, and tools of implementation science” [3]. Equity-oriented D&I research also occurs when evidence-based interventions that promote health equity or address health disparities and their root causes (e.g., structural racism) are routinely disseminated and implemented in clinical and community settings serving historically and systemically marginalized communities [1, 4].

This attention to promoting health equity has led to increased efforts in integrating principles from health disparities and D&I research with the goal of accelerating and enhancing translation of knowledge to improve health equity [1, 5]. Among health disparities researchers, there are growing opportunities for training in D&I research, such as Research in Implementation Science for Equity (RISE) Program (https://cvp.ucsf.edu/programs/capacity-building-training/research-implementation-science-equity-rise) and the Health Disparities Research Institute led by the National Institute for Minority Health and Health Disparities (https://www.nimhd.nih.gov/programs/edu-training/hdri/). Among D&I researchers, there has been recent interest and integration of health equity principles into frameworks [1, 4, 6, 7] and methods and measurement [8–10]; additionally, some D&I trainings have embedded an equity focus in their programs (e.g., The Institute for Implementation Science Scholars; IS-2).

The bidirectional learning and synthesis across these two fields, however, remains limited and siloed and thus, prevents the conduct of robust equity-oriented D&I research. Some challenges include the substantial underrepresentation of historically and systematically marginalized populations in research [1], limited development and testing of equity-oriented interventions to address underlying structural and social determinants of health [7], underdevelopment of equity-oriented measures and methods, inadequate attention to equity-relevant context, and misalignment of research processes with the needs of populations experiencing inequities [9]. Because research across all phases of the translational research pipeline, we must assume that majority of current D&I research does not adequately detect, understand, or improve upon disparities. Also, because researchers from both health disparities and D&I research often have little training in the complementary field, we must also assume that many people who could more comprehensively conduct equity-oriented D&I research have not reached their full potential to do such work and that training programs are likely not fully integrated yet to teach them.

Furthermore, the calls for integration of these two fields are largely theoretical and often focus on changes in applying frameworks [7] and methods [11], consideration for research questions [12], and how implementation processes are carried out and initiated [13, 14]. These foci require extensive information, skills, and collaborations across both fields (i.e., health disparities and D&I research) to develop and conduct impactful research. To accelerate integration of these fields, we need to better understand researchers’ perceived obstacles, drivers, and strategies to conduct equity-oriented D&I research. However, to date there have been no empirical inquiries about the needs of researchers to promote synergy and bi-directional learning across these fields. Learning from researchers about their perspectives regarding what is necessary to leverage and apply each fields’ contributions is a critical next step in cross-collaboration and promoting a focus on equity-oriented D&I research.

The purpose of this exploratory study was to understand current training needs by assessing among researchers their capability, opportunity, and motivation to conduct equity-oriented D&I research.

## Methods

### Theoretical approach

We used the Capability, Opportunity, and Motivation Model of Behavior Change (COM-B) to guide data collection, situate and organize results, and interpret findings [15]. To be able to engage in, develop, and conduct equity-oriented D&I research, researchers and their teams must have: 1) the Capability to psychologically and physically integrate equity-oriented in D&I research (i.e., knowledge, skills); 2) the Opportunity through external factors to make this change (i.e., environment, time, resources); and 3) the Motivation to do so, including reflective processes (e.g., making plans, evaluating changes already tried) and automatic processes (e.g., emotions). Because education and training are interventions that target behavior, and because COM-B has been used to design training (e.g., we believed this framework would be a good fit to help us understand potential behavior change mechanisms that could facilitate or hinder the conduct of equity-oriented D&I research [16, 17]. We hypothesized that the interaction of each factor is theoretically necessary to influence our decision-making and behaviors in learning, developing, and conducting equity-oriented D&I research.

### Study design

We conducted an exploratory cross-sectional survey that was distributed online from December 2020 to April 2021. This was an online survey, collected via Qualtrics survey platform [18]. This study was approved by the Institutional Review Board at Washington University of St. Louis (HRPO #202,012,103).

### Data description

#### Demographics and competencies

We asked about participants’ gender identity, race/ethnicity, whether they were an academic or practitioner (or other), and if academic, their academic role (i.e., faculty, staff, trainee). We also asked respondents to rate themselves as Novice, Advanced Beginner, Intermediate, or Advanced, informed from the literature describing and assessing D&I competencies. Similar to Padek and colleagues [19], we did not provide definitions for these terms to allow participants to self-define and prevent unintended bias from the research team on how they would define these different competency levels. For each competency level, we asked respondents to indicate their expertise in five different fields: D&I research, Health disparities research, Community Based Participatory Research (CBPR), Health-equity related research, D&I research with a focus on health equity.

#### Capability, opportunity, and motivation

We administered a total of 22 questions, developed by the authors, that collectively queried about the capacity, opportunity, and motivation towards engaging in developing and conducting equity-oriented D&I research. Eight questions about *Capability* assessed information and skills to complete tasks such as (1) applying theories, models, frameworks for promoting health equity in D&I research; (2) conducting a contextual assessment (i.e., formative evaluation, diagnostic assessment of the inequitable implementation problem) to inform methods to promote health equity in D&I research; (3) identifying evidence-based interventions to promote health equity in D&I research; and (4) defining and operationalizing or measuring health equity in D&I research. We also included two capability related statements in a question asking about the researcher’s ability to incorporate health-equity focus into D&I research (i.e., skills necessary to conduct this type of research). Capability questions were assessed on a Likert scale (“Neither Agree nor Disagree”; *Range* = *1 to 7*).

Similar to *Capability*, we queried about researchers’ *Motivations* towards the four tasks mentioned before, and an item asking whether researchers believe this type of research is needed for the field of D&I to move forward, under the question “What are some factors that could influence your ability to incorporate health equity into your D&I research”.

*Opportunity* was assessed using four questions focused on whether participants: (1) used any existing theories, models, and frameworks with an equity lens in their current D&I projects; (2) used any measures that incorporate an equity lens (e.g., measures of racism, community engagement) in their current D&I projects; (3) used any relevant measures that incorporate an equity lens for community engagement in their current projects; and (4) wanted training opportunities to accomplish the goal of incorporating health equity into your D&I research. The last set of questions were designed to query about training opportunities around specific domains and concepts in health equity and D&I. We queried about factors influencing their ability to incorporate health equity into D&I research and included three statements related to opportunity: (1) institutional support needed, (2) difficulties in receiving funding for equity-oriented D&I research, and (3) challenges in finding appropriate collaborators to engage in equity-oriented D&I research. Questions varied in their format and response options with the goal of capturing different aspects of these domains; the survey is available in Additional file 1.

### Sampling methods

We recruited participants at conferences (e.g., the 2020 D&I conference, hosted by the AcademyHealth), meetings, or listservs on either D&I or health disparities research (e.g., Division 45 [Society for the Psychological Study of Culture, Ethnicity, and Race] from the American Psychological Association, the Implementation Research Institute, National Implementation Research Network, Implementation Science Centers in Cancer Control). We also sent tweets and emails from our personal accounts to recruit participants. While we did not have eligibility inclusion criteria for recruiting, our emails and tweets explicitly invited participants to give us input on training needs with regards to health equity and implementation science, with the goal of understanding existing gaps and to develop future training opportunities in this space. The emails and tweets contained a link to the survey developed using the Qualtrics survey platform [18]. The consent form was provided at the start of the survey and described the purpose of the study and study team. Once consented, participants were able to answer the survey. To minimize bias in reporting, we did not collect any identifiable information about participants.

### Analysis

All analyses were conducted using SPSS Version 27.0. We calculated descriptive statistics including frequencies, means, and standard deviations and reported on all data from questions answered by participants. To assess differences in training opportunities to conduct equity-oriented D&I research by participants’ training level, we conducted an Analysis of Variance (ANOVA) with training level as the independent variable (i.e., trainees, faculty, other) and individual survey items assessing motivation, information, and skills as dependent variables. We used ANOVA as the diagnostic test given that we wished to compare means of the three groups and our outcome variables are continuous (i.e., based on aggregate responses to the survey questions). Fischer’s Chi-square test would not be appropriate for this analysis because it is used to compare group means when the outcome variables are categorical. After assessing overall effects using ANOVA, we conducted post hoc analyses to determine specific between-group differences using Tukey’s honestly significant difference test.

Given our focus on reporting only descriptive statistics and comparing only completed responses, Little’s Test of Missing Completely at Random [20] was not conducted and data were not imputed.

## Results

### Demographics and competencies

A total of 180 people answered the online survey. Most participants identified as women (81.7%), white (66.1%), academics (78.9%), and faculty members (53.9%). Additional participant characteristics are reported in Table 1. With regards to competencies, many participants reported they were ‘Advanced’ (36.7%) or ‘Advanced Beginners’ (27.8%) in the D&I field. Participants reported varying levels of expertise in health disparities research, community based participatory research (CBPR), and health equity. Many participants (37.8%) reported being novice to D&I research that focused on health equity. Table 2 provides the detailed descriptive statistics for respondents’ self-reported expertise in the five different categories of research (i.e., D&I Research, Health Disparities Research, Community Based Participatory Research, Health Equity Related Research, D&I Research with a Focus on Health Equity).

**Table 1:** Participant Demographics (*N* = 180)

Demographics		N (%)
Gender identity	Woman	147 (81.7%)
	Man	24 (13.3%)
	Non-binary	1 (0.6%)
	Prefer not to say	1 (0.6%)
	Did not respond	7 (3.8%)
Race and Ethnicity	American Indian or Alaska Native	2 (1.1%)
	Asian	23 (12.8%)
	Black, African American or African	20 (11.1%)
	Hispanic, Latino or Spanish	19 (10.6%)
	Middle Eastern or North African	4 (2.2%)
	Native Hawaiian or other Pacific Islander	0 (0%)
	White	119 (66.1%)
	None of these	3 (1.7%)
Academic experience	Academic	142 (78.9%)
	Practitioner	28 (15.6%)
	Other	21 (11.7%)
Current Role	Graduate student	28 (15.6%)
	Postdoctoral student	17 (9.4%)
	Faculty	97 (53.9%)
	Research Staff	21 (11.7%)
	Academic leadership	3 (1.7%)
	Other	15 (8.3%)

Responses may have varying percentages because participants were given the option to check all that apply

**Table 2:** Participants’ level of self-reported experience in fields related to D&I or health equity research

	Novice	Advanced Beginner	Intermediate	Advanced	Total N
D&I Research	20 (11.1%)	50 (27.8%)	44 (24.4%)	66 (36.7%)	180
Health Disparities Research	32 (17.8%)	35 (19.4%)	55 (30.6%)	47 (26.1%)	169
Community Based Participatory Research	41 (22.8%)	42 (23.3%)	49 (27.2%)	41 (22.8%)	173
Health Equity Related Research	39 (21.7%)	48 (26.7%)	39 (21.7%)	40 (22.2%)	166
D&I Research with a Focus on Health Equity	68 (37.8%)	49 (27.2%)	24 (13.3%)	22 (12.2%)	163

Responses may have varying percentages because participants were given the option to check all that apply

Below we report results of the discrete sets of questions. For each table, we provide the question and descriptive statistics based on the respective COM-B domain that informed the question.

Table 3 includes descriptive statistics, means, and standard deviations for questions related to capability and motivation for conducting equity-oriented D&I research. Regarding questions about knowledge or information, the overall mean of the four specific questions was 4.2, with participants responding to individual items assessing knowledge with a range of means from 3.99 (*SD* = 1.56) to 4.62 (*SD* = 1.51). The overall mean for questions that assessed necessary skills was 4.4, with participants responding to individual items with a range of means from 4.25 (*SD* = 1.76) = to 4.67 (*SD* = 1.17). Finally, the overall mean for questions assessing motivation was 6.2, with participants responding to individual items assessing motivation with a range of means from 5.97 (*SD* = 1.32) to 6.31 (*SD* = 1.03).

**Table 3:** Participants’ self-reported capacity and motivation for conducting equity-oriented D&I research

Questions	Strongly Disagree	Disagree	Some-what Disagree	Neither Agree Nor Disagree	Some-what Agree	Agree	Strongly Agree	Mean (SD)	N
Capability (Information)									
1. I have the information needed to apply theories, models, frameworks for promoting health equity in D&I research	13 (7.2%)	23 (12.8%)	24 (13.3%)	16 (8.9%)	62 (34.4%)	29 (16.1%)	9 (5.0%)	4.20 (1.67)	176
2. I have the information needed to conduct a contextual assessment (i.e., formative evaluation, diagnostic assessment of the inequitable implementation problem) to inform methods to promote health equity in D&I research	11 (6.1%)	31 (17.2%)	28 (15.6%)	20 (11.1%)	52 (28.9%)	25 (13.9%)	15 (8.3%)	4.14 (1.72)	182
3. I have the information needed to identify evidence-based interventions to promote health equity in D&I research	4 (2.2%)	18 (10.0%)	19 (10.6%)	28 (15.6%)	56 (31.1%)	34 (18.9%)	17 (9.4%)	4.62 (1.51)	176
4. I have the information needed to define and operationalize or measure health equity in D&I research	11 (6.1%)	25 (13.9%)	43 (23.9%)	24 (13.3%)	48 (26.7%)	20 (11.1%)	9 (5.0%)	3.99 (1.56)	180
Capability (Skills)									
1. I have the skills necessary to apply theories, models, frameworks for promoting health equity in D&I research	7 (3.9%)	20 (11.1%)	31 (17.2%)	15 (8.3%)	53 (29.4%)	37 (20.6%)	18 (10.0%)	4.50 (1.66)	181
2. I have the skills necessary to conduct a contextual assessment (i.e., formative evaluation, diagnostic assessment of the inequitable implementation problem) to inform efforts to promote health equity in D&I research	14 (7.8%)	26 (14.4%)	21 (11.7%)	22 (12.2%)	49 (27.2%)	30 (16.7%)	17 (9.4%)	4.25 (1.76)	179
3. I have the skills necessary to identify implementation strategies to promote health equity in D&I research	3 (1.7%)	16 (8.9%)	29 (16.1%)	22 (12.2%)	57 (31.7%)	34 (18.9%)	19 (10.6%)	4.67 (1.17)	180
4. I have the skills necessary to define and operationalize health equity in D&I research	8 (4.4%)	21 (11.7%)	30 (16.7%)	27 (15.0%)	50 (27.8%)	31 (17.2%)	11 (6.1%)	4.27 (1.60)	178
Motivation									
1. I am motivated to apply theories, models, frameworks for promoting health equity in D&I research	1 (0.6%)	1 (0.6%)	3 (1.7%)	6 (3.3%)	10 (5.6%)	42 (23.3%)	113 (62.8%)	6.41 (1.03)	176
2. I am motivated to conduct a contextual assessment (i.e., formative evaluation, diagnostic assessment of the inequitable implementation problem) to inform efforts to promote health equity in D&I research	2 (1.1%)	5 (2.8%)	3 (17.%)	13 (7.2%)	22 (12.2%)	55 (30.6%)	78 (43.3%)	5.97 (1.32)	178
3. I am motivated to identify implementation strategies to promote health equity in D&I research	2 (1.1%)	2 (1.1%)	3 (1.7%)	7 (3.9%)	11 (6.1%)	43 (23.9%)	110 (61.1%)	6.34 (1.17)	178
4. I am motivated to define and operationalize health equity in D&I research	1 (0.6%)	4 (2.2%)	1 (0.6%)	7 (3.9%)	19 (10.6%)	51 (28.3%)	98 (52.2%)	6.23 (1.14)	181

Table 4 provides an overview of participants' perceptions regarding the opportunities available to engage in, propose, and conduct equity-oriented D&I projects. When queried about their use of theories, models, or frameworks with an equity lens in current D&I projects, 68 (37.8%) reported using the RE-AIM extension for health equity and sustainability [21], 35 (19.4%) reported Health Equity Implementation Framework [6], and 25 (13.9%) reported using Baumann and Cabassa considerations for the Proctor Model [1]. Some examples of other frameworks participants reported using in the open text included the Conceptual Framework of the Multilevel Influence of Racism on Rural Cancer Disparities Across the Continuum [22], Consolidated Framework for Implementation Research [23], RE-AIM (without extension) [24], the Exploration, Preparation, Implementation, Sustainment framework [25], the Interactive Systems Framework [26], Theoretical Domains Framework [27] Black Feminist Thought [28], The World Health Organization’s Social Determinant of Health Framework [29], and Proctor’s Outcomes for Implementation Research framework [30] Interestingly, participants also mentioned research approaches (i.e., Community Based Participatory Research and Decolonizing Methodologies) as well as the Expert Recommendations for Implementing Change (ERIC) [31] as answers for this question.

**Table 4:** Participant’s response on the opportunities or external factors towards engaging, proposing, and conducting equity-oriented D&I research

A. Use of existing theories, models, and frameworks	N (%)
Health Equity Implementation Framework	35 (19.4%)
RE-AIM – extension for health equity and sustainability	68 (37.8%)
Baumann and Cabassa considerations for the Proctor Model	25 (13.9%)
Other D&I theories, models, frameworks applied with an equity lens. Please specify	33 (18.3%)
Other equity related theories, models, frameworks applied to D&I research. Please specify	22 (12.2%)
B. Types of measures	N (%)
Individual-level factors (i.e. racist bias, impact of sexual orientation and gender identity, etc.)	75 (41.7%)
Community-level factors (i.e. perceived structural racism scale, etc.)	58 (31.1%)
Healthcare setting-level factors (i.e. major experiences of discrimination, etc.)	43 (23.9%)
State and national policy level	24 (13.3%)
Other measures. Please specify	12 (6.7%)
C. Use of relevant measures that incorporate an equity lens for community engagement	N (%)
Yes	28 (15.6%)
D. Training participants reported wanting to accomplish the goal of incorporating health equity into D&I research	N (%)
Training towards the use of theories, models, frameworks in health equity	103 (57.2%)
Training towards the use of theories, models, frameworks in D&I research	68 (37.8%)
Training to help guide the assessment of context with a focus on health equity	111 (61.7%)
Training to help select and utilize implementation strategies to promote equity	138 (76.7%)
Training to help select appropriate evidence-based interventions or practices to promote equity	98 (54.4%)
Training to help operationalize health equity outcomes or determinants	119 (66.1%)
Training to conduct community engaged D&I research	84 (46.7%)
Training on anti-racism and addressing structural racism	94 (52.2%)
Other. Please specify	11 (6.1%)

Participants could check all that applied and percentages are based on overall sample size of 180

When asked about measures used, only 28 (15.6%) participants answered they used any measure that incorporated an equity lens (e.g., measures for community engagement, racism, or discrimination) in their current projects. Forty-one percent reported using individual-level measures to examine issues of equity (e.g., stigma measures, implicit attitudes tests), followed by 31.1% using community-level measures (e.g., perceived structural racism scale, etc.) and 23.9% using healthcare setting measures (e.g., major experiences of discriminations in life domains, such as at school, job, housing). When asked for more information about measures used to incorporate an equity lens in current D&I projects, participants reported using qualitative approaches or not using any validated or existing measures.

We also asked participants what training opportunities they would need to help them incorporate a focus on health equity in D&I research. Most (76.7%) needed training to help select and utilize implementation strategies to promote equity, 66.1% needed training about how to operationalize health equity outcomes or determinants, and 61.7% needed training to guide assessment of context with a focus on health equity. When asked whether they would need other types of trainings, participants mentioned in open text that they would like training on: “*everything health equity, D&I and intersectionality”,* on *“the global application of equity and D&I research”,* on *“how to navigate D&I and health equity where there is not an applicable evidence-based intervention”,* and on *“how to implement trauma informed care practices in healthcare settings.*” Participants reported wanting more training on tools for how to do equity work, and training about how to, as early career faculty, advocate for equity work with more senior scholars.

Table 5 shows ANOVA results with the training answers by different levels of participants’ training. Graduate students and postdoctoral students were combined into a “trainee” category (*n* = 45), “faculty” includes people who responded they were in faculty positions (*n* = 97), and the “other” category is comprised of leadership, research staff, and people who selected the other category (*n* = 39). Results indicated mean differences by participants’ training level (i.e., trainees, faculty, other). There were main effects on three items. There was a significant overall effect by participants’ training level on the item “I have the skills necessary to identify implementation strategies to promote health equity in D&I research”, although post hoc analysis showed no meaningful differences between groups. There was a significant overall effect by participants’ training level on the item, “I have the information needed to apply theories, models, frameworks for promoting health equity in D&I research” such that faculty reported meaningfully higher scores than trainees (*p* = 0.027) and other participants (*p* = 0.021). Third, there was a significant overall effect by participants’ training level on the item, “I have the information needed to identify evidence-based interventions to promote health equity in D&I research,” such that faculty indicated higher levels of information than trainees (*p* = 0.004).

**Table 5:** ANOVA results of training questions by participants’ training levels

	Predictor	Mean	SD	Sums of Squares	df	Mean Square	F	p
I have the skills necessary to apply theories, models, frameworks for promoting health equity in D&I research	Trainee	4.41	1.64	14.841	2	7.421	2.72	0.07
	Other	3.97	1.67					
	Faculty	4.72	1.65					
I have the skills necessary to conduct a contextual assessment (i.e., formative evaluation, diagnostic assessment of the inequitable implementation problem) to inform efforts to promote health equity in D&I research	Trainee	3.90	1.80	10.967	2	5.484	1.76	0.17
	Other	4.03	1.71					
	Faculty	4.46	1.77					
I have the skills necessary to identify implementation strategies to promote health equity in D&I research	Trainee	4.34	1.57	15.032	2	7.516	3.38	0.04*
	Other	4.29	1.58					
	Faculty	4.92	1.43					
I have the skills necessary to define and operationalize health equity in D&I research	Trainee	3.98	1.54	11.893	2	5.947	2.35	0.98
	Other	3.94	1.61					
	Faculty	4.49	1.60					
I have the information needed to apply theories, models, frameworks for promoting health equity in D&I research	Trainee	3.74	1.71	28.992	2	14.496	5.49	0.005**
	Other	3.68	1.72					
	Faculty	4.55	1.55					
I have the information needed to conduct a contextual assessment (i.e., formative evaluation, diagnostic assessment of the inequitable implementation problem) to inform methods to promote health equity in D&I research	Trainee	3.83	1.73	16.935	2	8.467	2.89	0.58
	Other	3.71	1.82					
	Faculty	4.40	1.66					
I have the information needed to identify evidence-based interventions to promote health equity in D&I research	Trainee	4.07	1.65	26.518	2	13.259	6.09	0.003*
	Other	4.31	1.39					
	Faculty	4.96	1.43					
I have the information needed to define and operationalize or measure health equity in D&I research	Trainee	3.73	1.53	12.102	2	6.051	2.56	0.08
	Other	3.60	1.56					
	Faculty	4.20	1.55					

^*^p < 0.05, ^**^p < 0.01

Table 6 includes responses to questions about factors that could influence participants’ ability to conduct equity-oriented D&I research. Forty-four percent of participants reported the challenge of not having skills needed to conduct this research, 32.2% reported challenges in receiving funding to support this work, and 30% reported challenges with finding collaborators.

**Table 6:** Factors that could influence your ability to incorporate health equity into D&I research

COM-B Domains	Factors	N (%)
C	I do not have the skills necessary to conduct this type of research	80 (44.4%)
O	I do not have the institutional support needed	23 (12.8%)
C	I don’t have the time to obtain the training needed	33 (18.3%)
O	It is difficult to receive funding for this type of research	58 (32.2%)
O	It is challenging to find appropriate collaborators to engage in these research areas	54 (30.0%)
M	I do not believe this type of research is needed for the field of D&I to move forward	0 (0.0%)
	Others	17 (9.4%)

Participants could check all that applied and percentages are based on overall sample size of 180. COM-B domains are *C* Capability, *M* Motivation, *O* Opportunity

Table 7 outlines specific challenges to funding (written into open text), including lack of “*funding that honors local partnership and time to appropriately tailor/adapt and implement interventions to address long standing structural factors that contribute to health inequities.*” Other reported barriers for conducting this type of research included “*lack of mentorship, hierarchy (e.g., staff cannot control research agenda), concerns that existing tools are created by white people, novelty or newness of the field,* and *lack of infrastructure or experience*.” All participants agreed that equity-oriented research is needed for advancing D&I research.

**Table 7:** Factors that could influence participants’ ability to incorporate health equity into their D&I research, as reported by open text

Available time to dedicate more focus to this work. I do have some work in this area
Baseline data collaborators don't find this important enough
Finding a mentor is actually very challenging. I have tried through [blinded], but I think they are perhaps overwhelmed and I know they have extreme challenges with their website. My mentor requests through their website have gone unnoticed, I think, over about 1 1/2 years. Other possibilities would be very welcome!
I am a staff at the mercy of investigators and how they choose to focus their research projects
I don't think we have the TMF, strategies, and measures to really do equity-focused D&I. We're just getting started as a field
I think the field is in its infancy in terms of application of health equity into D&I and as tools are available, I will use them
It is notable to me that many of the commonly cited health equity imp sci or health services/public health papers (as least noted on this webpage) are written by white scientists. I think the lack of representation of POC and Black scholars in imp sci makes me hesitant to get training in interventions for systemic racism from white investigators
None of these—skills, support, and collaborators are all available and this is a crucial topic
Sometimes it is the D&I researchers who resist this approach or exclude equity researchers as not really D&I
Sometimes labels create division- I am not specifically trained in D&I but the work I do is focused in the same way. There is a need to break down jargon
The care system in which one is engaged may not be ready or inclined to provide an infrastructure for this work. The very structural racism one may study operates to supress this very work
The models are a good start. The next step is framing/phrasing research proposals addressing this topic
There is a priority toward big data and large numbers. Equity focused work often happens in one community, one clinic at a time. We need better funding models that honors local partnership and time to appropriately tailor/adapt and implement interventions to address long standing structural factors that contribute to health inequities. Also, those who come to academia with a focus on inequities or CBPR are often socialized away from their prioritize in order to stay employed/funded/advance on the tenured track. "Do CBPR later in your career, it takes too long to get publications."
There is limited consideration of health equity within a lot of D&I research
Whether D&I funders see adapted interventions as “evidence based” — “marginalized” means populations which have been relegated to the margins of generalizability!
While important, this is not a lens I have typically applied

## Discussion

Participants in this study represent a range of experience levels, from novice to advanced, in D&I, health disparities, health equity, and community-based participatory research. Among respondents, there was a modest proportion (25.5%) who were intermediate or advanced in D&I research focused on health equity.

Our findings indicate that even though people were highly motivated to conduct equity-oriented D&I research, their capability in terms of knowledge was reported as a barrier to conducting this type of work. There was variability in terms of access to information: faculty participants tended to report higher levels of information to conduct equity-oriented D&I research than trainees, academic staff or research assistants, or practitioners. Several participants reported not having the information needed to conduct a contextual assessment to inform efforts to promote health equity in D&I research, or they did not know about existing frameworks focused on D&I and health equity. When asked about what types of training participants would like to receive, most participants reported needing training in how to assess context with a health equity focus, and on how to select strategies to promote equity. Measuring context is indeed a challenge for the D&I field because of its dynamic and complex characteristics [1, 6]. There are preliminary guides to measuring context in D&I research [32], with some focused on equity [7]. Yet, more work is needed to blend the wealth of knowledge between the fields of health equity and D&I to effectively develop best practices to examine context and develop strategies that enhance trust, collaboration, and equity [32–36].

One potential reason for respondents’ perceived barriers regarding capacity for conducting equity-oriented research could be the relatively new and untested adapted D&I theories, models, and frameworks with explicit foci on equity, even though literature on health equity with guidance relevant to D&I (e.g., community based participatory research) is not new [37, 38]. Most frameworks we queried about in our survey were predominantly from recent publications and have not been empirically piloted or tested, with exception of the Health Equity Implementation Framework [6, 7]. Scholars have adapted other frameworks, such as the Consolidated Framework of Implementation Research, adapted to incorporate a racism conscious aspect (e.g., [39]). It is worth noting that, as indicated by some of the open text responses, researchers are using theories, models, and frameworks from other disciplines such as anthropology, sociology, and ethics [14, 40], indicating there could be an integration of equity frameworks in their D&I work that does not necessarily involve adaptations of our existing traditional D&I frameworks, which is also appropriate.

One opportunity for engagement in equity-oriented D&I research was adequate funding. Participants reported difficulty in identifying and receiving funding for this type of research, difficulties in finding appropriate collaborators to engage, and/or not having necessary institutional support to conduct research in these areas. These barriers have been reported by other D&I researchers [9]. Specific examples of barriers for funding opportunity include inequalities in research funding for non-white, non-male researchers [41–44], limited opportunities to strengthen understanding of social determinants of health, systemic racism and discrimination in research and practice [4, 45–48], and unfavorable institutional rankings towards health disparities research compared to what is perceived as more innovative “-omics” research [45].

Other factors related to opportunities and motivation reported by participants that prevented their conduct of equity-oriented D&I research included: lack of mentorship, hierarchies in which they could not shape research agendas, concerns that existing tools are created by white people, and newness of integrating these fields. Mentorship and promotion to conduct equity-focused research is a challenge in many fields, including issues with a burdensome minority tax for faculty and staff with lived experience with inequities, and recruitment and retention of researchers with lived experiences and expertise as equity-oriented D&I research trainees and faculty [46, 49]. One implication for hiring institutions is to effectively include historically and systematically marginalized groups, including women, Black scholars, indigenous people, people of color, people with disabilities, non-binary people, and their allies [50, 51] in promotion packages by extending the metrics of success beyond publication and citation metrics.

Based on our exploratory findings, we join others in advocating that training in equity-oriented D&I is more than “talking about” or describing equity issues or health inequities, and should strengthen capacity through increase in knowledge, skills, and reflection in “how to” take action to address issues contributing to disparities, such as racism, discrimination, and poverty [4, 52, 53], and doing so with care and community input because reactive action can impose more harm than benefit. As such, we propose a multi-pronged approach for increasing equity-oriented D&I research: increase in a) opportunities for training in equity-oriented D&I, b) collaboration between D&I researchers with individuals with expertise and lived experience with health equity research, c) funding for equity-oriented D&I research, and d) recognition of the value of community engaged research in promotion packages. We hypothesize that enhancing capability through training, as well as increasing external opportunities through more funding, networking with potential collaborators, and mentorship and promotion packages specifically focused on health equity will be essential next steps to building capacity for equity-oriented D&I research [9, 54, 55].

First, we need to increase opportunities for training and improve access to such trainings for all types of learners, to enhance equity-oriented D&I knowledge and skills for people with health disparities, equity, or D&I backgrounds. Although some D&I trainings have an equity lens and some health equity trainings have components of D&I, a training developed with a clearer intention of integrating these fields, led by diverse researchers and practitioners, would be beneficial to all and provide opportunities for novel advancements and innovations across these fields.

A challenge in developing such training lies in the very problem of equity: how to increase the reach of a training program and provide quality mentoring with limited funding currently provided to such programs. Short-term solutions, such as freely available videos or modules, may help increase reach (e.g., [56, 57]). Long-term solutions would include a seismic shift in funding to support this type of training, funding smaller institutions, and funding recruitment, training, and retention of a diverse workforce (including those with lived experience with inequities) to support different career paths and strengthen diversity and value in research teams [58–60].

Second, we need to thoughtfully collaborate with individuals with expertise and lived experience with health inequities in D&I and health equity research fields. In the academic context, this will require deeper reflection and accountability processes for addressing systemic racism within academic institutions [61] to be able to recruit, train, and retain scientists of historically marginalized backgrounds to lead and collaborate on research and implementation. Data has shown that scholars of color, scholars of varying levels of ability, and others scholars with lived experience of racism and discrimination are often not recruited and, if recruited, are often not retained in academia [58, 59]. Third, participants mentioned lack of explicit funding announcements for equity-oriented D&I research, such as incentives that value cultural adaptation, longer timelines, and mixed methodologies. Community engagement in research, as one method to ground and address disparities, necessitates funding with a larger financial cap for community organizations, ensuring high financial readiness for grant funds for community organizations, and encourage community members or leaders to be grant co-investigators, increasing the likelihood they have equal input and decision-making abilities, applicable financial compensation, and power [61–64], because academic fiscal practices may be a major barrier for community engaged, health equity focused work [65]. Community engaged research is the cornerstone of equity-oriented research and is not traditionally recognized in academic tenure, non-tenure, and promotion packages [66–68]. We need a fundamental shift in recognizing the value of community engaged research to reconsider other impacts of our work besides numbers of grants, dollars, and publications if we are to institutionalize equity as foundational in D&I research [50].

### Limitations

This study has some limitations. This is a cross-sectional, online survey that used newly developed questions not psychometrically validated. Given the paucity of empirical work in this area, we consider this to be an exploratory study in reaching a sample of researchers interested in these topic areas through meetings and social media. This exploratory attitude also allowed us to develop questions that were aimed at understanding researchers’ engagement with and conduct of equity-oriented D&I research. The sample of 180 participants is small and there may be sampling bias introduced through our sampling methods (e.g. underrepresenting those not active on social media or in professional organizations). Despite these limitations, this is, from our perspective, an innovative study in empirically exploring the perspective of participants from different backgrounds about factors that influence their ability to conduct equity-focused D&I research. The sample size for our study is comparable, if not larger, to the sample size of other survey studies used to inform D&I trainings (e.g., [69–71]. This survey did not assess faculty ranks (i.e., Assistant, Associate, Full Professor) which may have given further insight into how these challenges could perhaps be different for early career compared to more established faculty. Our questions about expertise on the different fields was “select all”, making it difficult to disentangle unique challenges per discipline. While we had informal conversations with international collaborators who stated that they answered our survey, we did not capture the geographical location of our participants and hence, are unable to highlight global participation. Our question about the use of frameworks was very specific to our own work. As shown by the open responses from our participants, there is a large literature about health equity in public health, global health and other social science literatures. The development of training in equity-oriented D&I research should bring the broader literature and examine how these different disciplines could strengthen the work around equity through collaboration. Finally, participants could have also represented several topic areas, such as cancer or mental health, and therefore, have differential exposure to equity or D&I research based on topic interest.

## Conclusions

To the best of our knowledge, this is the first empirical paper on barriers for researchers to conduct equity-oriented D&I research, highlighting that although participants have great motivation to do the work, they need further training in how to conduct this work. Specific reported needs were around increasing capability (e.g., knowledge about and skills on how to apply frameworks and measures), and opportunities (e.g., funding that prioritizes equity work, and infrastructure to facilitate networking with collaborators) Based on these results, we advocate for an increase in opportunities for training specifically in equity-oriented D&I, collaboration between D&I researchers with individuals with expertise and lived experience with health equity research, funding for equity-oriented D&I research, and recognition of the value of community engaged research in promotion packages.

## Supplementary Information

**Additional file 1.**

## Data Availability

All the data used in the study was made provided to corresponding author on a reasonable request.

## Abbreviations

ERIC: Expert Recommendations in Change
D&I: Dissemination and implementation
CBPR: Community Based Participatory Research
COM-B: Capability, Opportunity, Motivation Model
