# Supplementary material for: Assessing researchers’ capabilities, opportunities, and motivation to conduct equity-oriented dissemination and implementation research, an exploratory cross-sectional study
Source: BMC Health Serv Res. 2022 Jun 1;22:731. doi: 10.1186/s12913-022-07882-x (PMC9161573; doi:10.1186/s12913-022-07882-x)
Supplement: Supplementary file 1 — Additional file 1. [file 12913_2022_7882_MOESM1_ESM.docx]

**Appendix 1 – Survey**

Equity and Implementation Science

This survey aims to capture future needs for training competencies essential for conducting implementation science with a health equity focus.

For the purposes of this survey, we consider health disparities to be differences in health outcomes that are due to social injustices [1]. We consider health equity as the "Principle underlying a commitment to reduce, and ultimate, eliminate disparities in health and its determinants, including social determinants" [2].

 All of the data collected in this survey is anonymous and will be used to guide the development of future training activities. 
 
  [1] When Does A Difference Become A Disparity? Conceptualizing Racial And Ethnic Disparities In Health
 Paul L. Hebert, Jane E. Sisk, and Elizabeth A. Howell; Health Affairs 2008 27:2, 374-382

[1] Braveman P. What are health disparities and health equity? We need to be clear. Public Health Rep. 2014;129 Suppl 2(Suppl 2):5-8. doi:10.1177/00333549141291S203

**Please indicate your level of experience with the following research foci (Check all that apply).**

Answers: D&I research, health disparities research, community based participatory related research, and D&I research with a focus on health equity.

Questions:

|  |
| --- |
| I am a novice (I have not engaged in any activities related to this research field) (1) |
| I have advanced beginner research skills (e.g., I have participated in some training activities and may have contributed to a proposal/ project) (2) |
| I have intermediate research skills (I have engaged in some projects in the past but have not led a proposal/project with the research area as its main focus) (3) |
| I have advanced research skills in this field (I have led grant(s)/project(s) within this research field) (4) |

**Please indicate your agreement with the following statements:**

Answers: Likert Scale: Strongly Disagree, Disagree, Somewhat Disagree, Neither Agree nor Disagree, Somewhat Agree, Agree, Strongly Agree.

| Questions |
| --- |
| I have the information needed to apply theories, models, frameworks for promoting health equity in D&I research (1) |
| I am motivated to apply theories, models, frameworks for promoting health equity in D&I research (2) |
| I have the skills necessary to apply theories, models, frameworks for promoting health equity in D&I research (3) |
| I have the information needed to conduct a contextual assessment (i.e., formative evaluation, diagnostic assessment of the inequitable implementation problem) to inform methods to promote health equity in D&I research (4) |
| I am motivated to conduct a contextual assessment (i.e., formative formative evaluation, diagnostic assessment of the inequitable implementation problem) to inform efforts to promote health equity in D&I research (5) |
| I have the skills necessary to conduct a contextual assessment (i.e., formative evaluation, diagnostic assessment of the inequitable implementation problem) to inform efforts to promote health equity in D&I research (6) |
| I have the information needed to identify evidence-based interventions to promote health equity in D&I research (7) |
| I am motivated to identify implementation strategies to promote health equity in D&I research (8) |
| I have the skills necessary to identify implementation strategies to promote health equity in D&I research (9) |
| I have the information needed to define and operationalize or measure health equity in D&I research (10) |
| I am motivated to define and operationalize health equity in D&I research (11) |
| I have the skills necessary to define and operationalize health equity in D&I research (12) |

**Have you used any of the following theories, models, or frameworks with an equity lens in your current D&I projects?**

Answers: Yes/No/I am not aware of this framework

|  |
| --- |
| - Health equity implementation framework (1) |
| - REAIM – extension for health equity and sustainability (2) |
| - Baumann and Cabassa considerations for the Proctor Model (3) |
| - Other D&I theories, models, frameworks applied with an equity lens. Please specify: (4) |
| - Other equity related theories, models, frameworks applied to D&I research. Please specify: (5) |

**Have you used any** **measures that incorporate an equity lens (i.e. measures of racism, community engagement) in your current D&I projects? (check all that apply)**

- Individual-level factors (i.e. racist bias, impact of sexual orientation and gender identity, etc.) (1)
- Community-level factors (i.e. perceived structural racism scale, etc.) (2)
- Healthcare setting-level factors (i.e. major experiences of discrimination, etc.) (3)
- State and national policy level (4)
- Other measures used (5)

**Have you** **used any relevant measures that incorporate an equity lens for community engagement in your current projects?**

Answers: Yes (please specify)/No

**What** **training opportunities would you personally want to accomplish the goal of incorporating health equity into your D&I research?**

- Training towards the use of theories, models, frameworks in health equity (1)
- Training towards the use of theories, models, frameworks in D&I research (2)
- Training to help guide the assessment of context with a focus on health equity (3)
- Training to help select and utilize implementation strategies to promote equity (4)
- Training to help select appropriate evidence-based interventions or practices to promote equity (6)
- Training to help operationalize health equity outcomes or determinants (7)
- Training to conduct community engaged D&I research (8)
- Training on anti-racism and addressing structural racism (9)
- Other (10)

**What are some factors that could influence your ability to incorporate health equity into your D&I research? (check all that apply)**

| - I do not have the skills necessary to conduct this type of research (1) - I do not have the institutional support needed (2) - I don’t have the time to obtain the training needed (3) - It is difficult to receive funding for this type of research (4) - It is challenging to find appropriate collaborators to engage in these research areas (5) - I do not believe this type of research is needed for the field of D&I to move forward (6) - Others: (7) |
| --- |

**This section asks for information about you and will be used to the extent that it helps us understand your perspectives better. All information will be aggregated so no individual respondent is identified. What is your current gender identity?**

- Woman (1)
- Man (2)
- Non-binary / third gender (3)
- Prefer not to say (4)

**Which categories describe you?**

- American Indian or Alaska Native (For example: Aztec, Blackfeet Tribe, Mayan, Navajo Nation, Native Village of Barrow Inupiat Traditional Government, Nome Eskimo Community, etc.) (1) (1)
- Asian (For example: Asian Indian, Chinese, Filipino, Japanese, Korean, Vietnamese, etc.) (2) (2)
- Black, African American or African (For example: African American, Ethiopian, Haitian, Jamaican, Nigerian, Somali, etc.) (3) (3)
- Hispanic, Latino or Spanish (For example: Colombian, Cuban, Dominican, Mexican or Mexican American, Puerto Rican, Salvadoran, etc.) (4) (4)
- Middle Eastern or North African (For example: Algerian, Egyptian, Iranian, Lebanese, Moroccan, Syrian, etc.) (5) (5)
- Native Hawaiian or other Pacific Islander (For example: Chamorro, Fijian, Marshallese, Native Hawaiian, Tongan, etc.) (6) (6)
- White (For example: English, European, French, German, Irish, Italian, Polish, etc.) (7) (7)
- None of these fully describe me. (8)

**Do you consider yourself to be:**

- An academic (1)
- A practitioner (2)
- Other (3)

**What is your current role?**

- Graduate student (1)
- Postdoctoral student (2)
- Faculty (3)
- Research staff (e.g., research coordinator) (4)
- Academic leadership (e.g., Dean, Provost) (5)
- Other (6)

**Thank you for answering our questions. How did you hear about this survey? (check all that apply)**

- twitter
- email
- other

**Appendix 2 – STROBE GUIDELINES**

|  | **Item No** |  |
| --- | --- | --- |
| **Title and abstract** | 1 | Title, Abstract |
| Introduction |  |  |
| Background/rationale | 2 | Pg. 6-7 |
| Objectives | 3 | Pg.7 |
| **Methods** |  |  |
| Study design | 4 | Title page, Abstract, Methods section |
| Setting | 5 | Pg.8 |
| Participants | 6 | Pg.8 |
| Variables | 7 | Pg.9-10 |
| Data sources/ measurement | 8* | Pg.9-10 |
| Bias | 9 | Pg.9 |
| Study size | 10 | Pg.11 |
| Quantitative variables | 11 | Pg.10-11 |
| Statistical methods | 12 | Pg.10-11 |
| **Results** |  |  |
| Participants | 13 | Pg. 11 |
| Descriptive Data | 14 | Pg.11 |
| Outcome Data | 15 | Pg. 11-16 |
| Main Results | 16 | Pg. 11-16 |
| Other analyses | 17 | n/a |
| **Discussion** |  |  |
| Key results | 18 | Pg. 15 |
| Limitations | 19 | Pg. 20 |
| Interpretation | 20 | Pg. 20 |
| Generalisability | 21 | Pg. 20 |
| **Other information** |  |  |
| Funding | 22 | Pg. 22 |
